# Long-Term Biochemical and Cardiovascular Profiles 3–6 Years After Preeclampsia: Impact of Angiogenic Imbalance During Pregnancy

**DOI:** 10.3390/jcm14238389

**Published:** 2025-11-26

**Authors:** Noah Costa, Judit Platero, Pablo Garcia-Manau, Olga Sanchez-Garcia, Clàudia Pellicer, Mariona Jordi, Zoraida Garcia, Carmen Garrido-Gimenez, Johana Ullmo, Madalina Nan, Josefina Mora, Alvaro Garcia-Osuna, Marta Choliz, Monica Cruz-Lemini, Maria del Carmen Medina, Elisa Llurba

**Affiliations:** 1 Institut de Recerca Sant Pau (IR SANT PAU), Sant Quintí 77-79, 08041 Barcelona, Spain; ncosta@santpau.cat (N.C.); jplaterod@santpau.cat (J.P.); osanchezg@santpau.cat (O.S.-G.); zoraidagarciaruiz@gmail.com (Z.G.); cgarrido@santpau.cat (C.G.-G.); jullmo@santpau.cat (J.U.); mcruzl@santpau.cat (M.C.-L.); mmedinam@santpau.cat (M.d.C.M.); ellurba@santpau.cat (E.L.); 2 Spanish Network in Maternal, Neonatal, Child and Developmental Health Research (RICORS-SAMID, RD24/0013/0001), Primary Care Interventions to Prevent Maternal and Child Chronic Diseases of Perinatal and Developmental Origin Network (RICORS-SAMID, RD21/0012/0001), Instituto de Salud Carlos III, 28029 Madrid, Spain; jmora@santpau.cat; 3 Departament de Pediatria, Obstetrícia i Ginecologia i de Medicina Preventiva i Salut Pública, Universitat Autònoma de Barcelona, 08193 Bellaterra, Spain; mcholiz@santpau.cat; 4 Maternal-Fetal Medicine Unit, Department of Obstetrics and Gynecology, Hospital de la Santa Creu i Sant Pau, Universitat Autònoma de Barcelona, Sant Antoni Maria Claret 167, 08025 Barcelona, Spain; cpellicer@santpau.cat (C.P.); mjordi@santpau.cat (M.J.); 5 Primary Care Interventions to Prevent Maternal and Child Chronic Diseases of Perinatal and Developmental Origin Network (RICORS-SAMID, RD21/0012/0019), Instituto de Salud Carlos III, 28029 Madrid, Spain; 6 Department of Clinical Biochemistry, Hospital de la Santa Creu i Sant Pau, Institut d’Investigació Biomèdica Sant Pau (IIB Sant Pau), 08041 Barcelona, Spain; mnan@santpau.cat (M.N.); agarciao@santpau.cat (A.G.-O.); 7 Department of Biochemistry and Molecular Biology, Facultat de Biociències, Universitat Autònoma de Barcelona, 08193 Barcelona, Spain

**Keywords:** preeclampsia, sFlt-1, PlGF, cardiovascular risk, endothelial dysfunction, metabolic profile, biomarkers

## Abstract

**Background/Objectives:** Preeclampsia is associated with long-term cardiovascular and metabolic risks. This study aimed to evaluate metabolic and cardiovascular biochemical profiles in women with a history of preeclampsia and angiogenic imbalance during pregnancy. **Methods:** We conducted a cross-sectional study at Hospital de la Santa Creu i Sant Pau between August 2023 and July 2025. Participants had been prospectively enrolled during pregnancy (2018–2022) and were re-evaluated 3 to 6 years later. Blood and urine samples were collected after a 12-h fast to assess hematological, metabolic, and cardiovascular markers. Angiogenic profiles were determined using sFlt-1/PlGF ratios obtained during pregnancy. Multivariable linear regression models were used to assess associations with a history of PE and angiogenic imbalance, adjusting for relevant confounders. **Results:** 363 participants were included. 113 (31.1%) had a history of preeclampsia. Women with previous preeclampsia showed slightly higher high-sensitivity troponin T concentrations [4.0 (3.0–6.0) ng/L vs. 3.2 (3.0–5.0) ng/L, *p* = 0.03]. Women with sFlt-1/PlGF ≥38 exhibited significantly higher urinary protein [0.09 (0.07–0.18) g/L vs. 0.08 (0.07–0.13) g/L, *p* = 0.01], potassium [4.25 (4.07–4.40) mmol/L vs. 4.19 (4.02–4.37) mmol/L, *p* = 0.048], and LDH concentrations [168 (150–189) U/L vs. 163 (149–177) U/L, *p* = 0.046], and lower leukocyte counts [6150 (5348–7055) vs. 6250 (5430–7450) U/mL, *p* = 0.03]. **Conclusions:** Women with angiogenic imbalance during pregnancy display subtle alterations in renal and endothelial function markers years after delivery, whereas those with preeclampsia show slightly higher troponin concentrations. These findings, though clinically irrelevant, suggest that pregnancy-related vascular dysfunction may have different long-term manifestations depending on whether the maternal cardiovascular system was sufficiently compromised to develop overt preeclampsia.

## 1. Introduction

Cardiovascular disease remains the leading cause of death among women worldwide, surpassing all other causes across the lifespan [1]. Pregnancy represents a unique physiological “stress test” for the maternal cardiovascular system, requiring profound hemodynamic, vascular, and metabolic adaptations to support fetal growth and placental perfusion. Among pregnancy complications, preeclampsia (PE) is a multisystem disorder characterized by new-onset hypertension after 20 weeks of gestation, accompanied by proteinuria and/or evidence of maternal organ or uteroplacental dysfunction [1,2]. Affecting approximately 2% to 4% of pregnancies, it contributes to over 70,000 maternal and 500,000 fetal deaths globally each year, making it a major cause of pregnancy-related morbidity and mortality [3,4].

The exact mechanisms underlying PE remain incompletely understood, yet it is widely recognized as a multifactorial disorder in which endothelial dysfunction represents a central pathophysiological feature [3]. Abnormal placentation seems the initial event, leading to the release of antiangiogenic and proinflammatory factors, particularly soluble fms-like tyrosine kinase-1 (sFlt-1), into the maternal circulation [5,6]. This response reduces placental growth factor (PlGF) levels and thus increasing the sFlt-1/PlGF ratio, leading to angiogenic imbalance and widespread vascular injury. Importantly, this imbalance can often be detected weeks before the onset of clinical symptoms of the disease [7,8].

Although PE is considered a pregnancy-specific disorder, it is well established that affected women face a significantly increased risk of developing cardiovascular disease later in life, with persistent alterations in myocardial function and adverse cardiac remodeling documented years after the index pregnancy [9,10]. In addition, women with a history of PE frequently show adverse biochemical profiles, including dyslipidemia, insulin resistance, and chronic low-grade inflammation, which may contribute to their increased cardiovascular risk [9,10]. In recent years, growing evidence has also linked angiogenic imbalance to future cardiovascular risk, and an elevated sFlt-1/PlGF ratio during pregnancy has been associated with increased cardiovascular morbidity for up to 12 years postpartum [11,12,13], and it have emerged as potential indicator of subclinical endothelial dysfunction and long-term cardiovascular vulnerability. Understanding the relationship between pregnancy-specific cardiovascular adaptation and future cardiovascular and metabolic risk is therefore critical to improving early identification and prevention strategies for women’s health. Thus, the aim of the study was to assess cardiovascular and metabolic biochemical profiles 3 to 6 years postpartum in women with a history of PE and to explore the potential impact of angiogenic imbalance during pregnancy on these long-term outcomes.

## 2. Materials and Methods

This was a cross-sectional carried out at Hospital de la Santa Creu i Sant Pau (Barcelona, Catalonia, Spain) between August 2023 and July 2025. The study population consisted of women prospectively enrolled during their index pregnancy as part of previous studies conducted from 2018 to 2022, (EUROPE, ANGIOCOR and BiSC) [14,15,16]. BiSC was a low-risk pregnancy cohort designed to comprehensively evaluate the socio-environmental and genetic determinants of fetal and child health, as well as maternal physical and mental wellbeing during and after pregnancy. EUROPE included women with suspected preeclampsia and aimed to assess the added value of incorporating the sFlt-1/PlGF ratio into clinical management. ANGIOCOR focused on the cardiovascular assessment of women at high risk of preeclampsia.

Inclusion criteria for the present follow-up study were participation in one of these three cohorts and a delivery 3–6 years prior to the follow-up visit. Exclusion criteria included unwillingness to participate or inability to provide informed consent. All eligible women were invited to participate in order to maximize representativeness and statistical power. These studies gathered detailed clinical information on maternal medical history and pregnancy-related complications. During pregnancy, serum levels of PlGF and sFlt-1 (pg/mL) were assessed using automated electrochemiluminescence immunoassays on the Roche Cobas^®^ e411 platform (Roche Diagnostics GmbH, Mannheim, Germany). These biomarkers were measured irrespective of the development of clinical signs suggestive of placental dysfunction, as specified in the protocols of the three studies. All analyses were performed in the same laboratory using identical methods to minimize inter-assay variability. Due to logistical limitations, the sFlt-1/PlGF ratio could not be obtained in all participants. When available, the closest value to delivery was used. Ratio values were classified into four groups (<38, ≥38 to <85, ≥85 to <110, and ≥110), according to the established cut-off values derived using the same analytical platform, as previously described to define angiogenic imbalance in PE [17]. PE was defined, according to the guidelines of the American College of Obstetricians and Gynecologists, as new-onset hypertension or worsening of preexisting hypertension during pregnancy, accompanied by proteinuria (protein/creatinine ratio of 0.3 mg/dL or more or dipstick reading of 2+ if quantitative method was not available) or evidence of maternal organ dysfunction, either clinical or laboratory-based. Organ dysfunction criteria included: thrombocytopenia (platelet count < 100 × 10^9^/L), renal insufficiency (serum creatinine ≥ 1.1 mg/dL or a doubling of baseline values in the absence of underlying renal disease), hepatic dysfunction (elevated liver transaminases to twice normal concentration), epigastric or right upper quadrant pain unresponsive to analgesia, pulmonary edema, new-onset headache unresponsive to treatment and not explained by alternative diagnoses, or visual disturbances such as blurred vision or photopsia [18]. All cases diagnosed with PE were cross-checked to confirm the diagnosis.

Participants were re-assessed between 3 and 6 years after their index pregnancy, after providing written informed consent approved by the Ethics Committee of the same institution (IIBSP-MOM-2022-87) on 19 July 2023. All procedures described refer to this postpartum visit. At this assessment, current maternal demographic characteristics and medical history were recorded. Blood pressure was measured once in a single arm, with the participant in a seated position, after a 5-min rest period, using an automated sphygmomanometer.

Peripheral blood samples from all participants in the study were obtained upon inclusion following a minimum fasting period of 12 h. For the assessment of proteinuria, the first morning void urine sample was collected and analyzed. Whole blood samples were collected by venipuncture in Vacutainer™ tubes (Becton Dickinson, Franklin Lakes, NJ, USA) and fractionated by centrifugation at 3000× *g* for 15 min at room temperature to obtain serum, which was aliquoted and stored at −80 °C until analyzed.

Routine hematological and biochemical variables, including lipid profile, were measured by standard, fully automated methods. Urine analyses assessed total protein concentration and albumin levels. In addition, thyroid-stimulating hormone and prolactin levels were evaluated. Serum concentrations of PlGF, N-terminal pro B-type natriuretic peptide, and high sensitivity Troponin T (hs-TnT) were also measured using automated electrochemiluminescence immunoassays on the Roche Cobas^®^ e601 platform (Roche Diagnostics GmbH, Mannheim, Germany). The measuring ranges were 3–10,000 pg/mL for PlGF, 10–35,000 pg/mL for NT-ProBNP and 3–10,000 ng/L for hs-TnT. Normal reference ranges for the hematological, biochemical and cardiovascular biomarker parameters are specified on Table S5.

### Statistical Analyses

Continuous variables were summarized using median values and interquartile ranges (IQR), while categorical variables were reported as absolute counts and corresponding percentages. The Shapiro–Wilk test was employed to evaluate the normality of data distributions. Group comparisons were conducted using the Mann–Whitney U-test or Student’s *t*-test for quantitative variables, while the chi-square test or Fisher’s exact test was used for categorical variables. For analyses involving more than two groups, the Kruskal–Wallis test was applied, followed by the Dwass–Steel–Critchlow–Fligner post hoc test for pairwise comparisons.

To explore the relationship between a history of PE in the index pregnancy and the outcomes of interest, multivariable linear regression analyses were conducted. Adjustment was made for variables that significantly differed between groups in univariable analyses, as these were considered potential confounders that could influence the association between exposure and outcome. This approach aimed to minimize overfitting while ensuring control for relevant baseline imbalances. An additional subanalysis comparing participants with early-onset PE (diagnosed < 34 weeks of gestation) versus late-onset PE (diagnosed ≥ 34 weeks of gestation) was performed. All statistical procedures were performed using Jamovi software (The Jamovi Project, Version 2.3, https://www.jamovi.org). A *p*-value < 0.05 (two-tailed) was considered indicative of statistical significance. The study adhered to the STROBE (Strengthening the Reporting of Observational Studies in Epidemiology) guidelines throughout its design and reporting.

## 3. Results

During the study period, 363 participants were included. Among them, 113 (31.1%) had a history of previous PE, while 250 (68.9%) did not. Women with a prior history of PE were slightly older and had a higher BMI compared to those without previous PE. Both current and pre-existing chronic hypertension were significantly more frequent among women with prior PE in the index pregnancy, who also exhibited higher systolic, diastolic, and mean arterial blood pressure values at study inclusion. Women with prior PE had worse anti-angiogenic profile during pregnancy (elevated sFlt-1, reduced PlGF, and higher sFlt-1/PlGF ratios). Other baseline and metabolic characteristics did not differ significantly between groups. Detailed characteristics are summarized in Table 1.

Participants with previous PE showed lower levels of low-density lipoprotein (LDL) compared to those without history of PE in the index pregnancy [111.0 (93.3–132.0) vs. 104.0 (93.0–130.0) mg/dL, *p* = 0.03]. No more differences were observed between groups regarding hematological or biochemical parameters, including those obtained from urine samples. Regarding cardiovascular biomarkers, hs-TnT levels were significantly higher in participants with a history of PE compared to those without [3.20 (3.00–5.00) vs. 4.00 (3.00–6.00) ng/L, *p* = 0.03], as shown in Table 2. No statistically significant differences were observed in PlGF and NT-proBNP concentrations.

Participants were also classified according to angiogenic imbalance during gestation (sFlt-1/PlGF < 38 or ≥38). sFlt-1 and PlGF values during gestation were available for 295 (81, 3%) participants. Of these, 213 women (72.2%) had a normal angiogenic profile (sFlt-1/PlGF < 38), while 82 (27.8%) showed abnormal angiogenic values (sFlt-1/PlGF ≥ 38). According to this classification, those participants with elevated sFlt-1/PlGF (≥38) exhibited higher BMI, a greater frequency of chronic hypertension, and higher blood pressure levels. Primiparity was also more common in this group. sFlt-1 and PlGF values and gestational age at sFlt-1/PlGF determination also differed between groups (Table 3).

As shown in Table 4, participants with an abnormal angiogenic profile during gestation (sFlt-1/PlGF ≥ 38) showed significantly higher urinary protein [0.08 (0.07–0.13) vs. 0.09 (0.07–0.18) g/L, *p* = 0.01], potassium [4.19 (4.02–4.37) vs. 4.25 (4.07–4.40) mmol/L, *p* = 0.048], LDH concentrations [163 (149–177) vs. 168 (150–189) U/L, *p* = 0.046], and lower leukocyte counts [6250 (5430–7450) vs. 6150 (5348–7055) U/L, *p* = 0.03] compared to those with normal sFlt-1/PlGF values. No significant differences were observed in other hematological, biochemical, or cardiovascular biomarkers between groups.

Among participants with a history of PE, 41 (36.3%) and 72 (63.7%) had a history of early-onset and late-onset PE, respectively. No statistically significant differences were found among the groups in hematological or biochemical parameters. Similarly, cardiovascular biomarkers did not differ between groups, although a trend towards higher hs-TnT levels was noted in the early-onset PE group (Tables S1 and S2).

**Figure 1:**
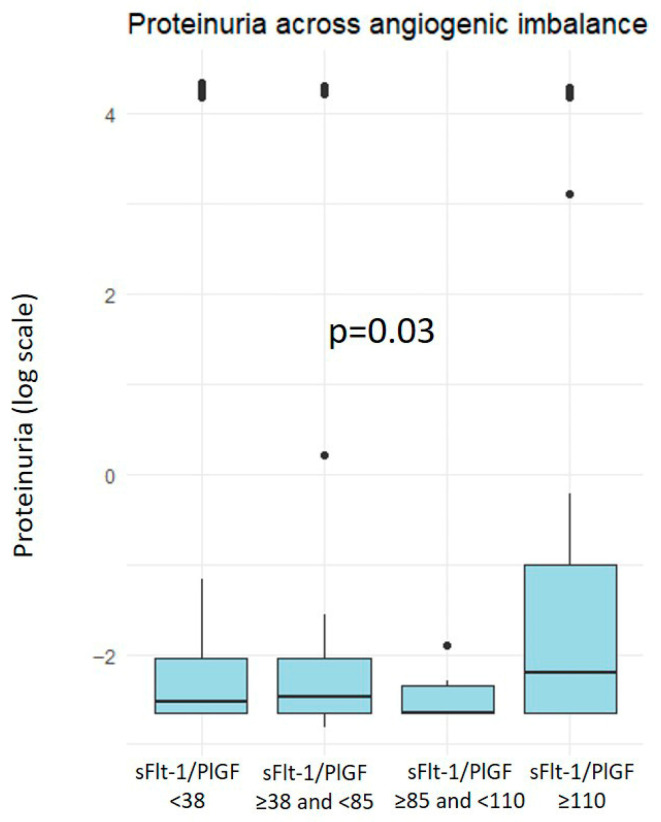
Boxplot showing urinary protein concentrations (log-scale) across groups defined by angiogenic imbalance during pregnancy, according to the sFlt-1/PlGF ratio.

Additionally, a subgroup analysis was performed among women with an abnormal angiogenic profile, further stratifying them into three categories: ≥38 and <85, ≥85 and <110, and ≥110. Significant differences were found in urinary protein concentrations and hematocrit values across the subgroups (Figure 1). More details can be found in Tables S3 and S4.

## 4. Discussion

### 4.1. Main Findings

An abnormal sFlt-1/PlGF ratio during pregnancy is associated with mild increases in urinary protein, potassium and LDH concentrations at 3–6 years postpartum and a mild decrease in total leukocytes count. The association with the degree of angiogenic imbalance is observed specifically for proteinuria, as greater imbalance corresponds to higher protein excretion. These findings may be related to persistent endothelial damage. In contrast, a history of PE is associated with slightly higher hs-TnT and lower LDL concentrations, while no differences are observed in proteinuria or LDH levels. These results, though clinically irrelevant, may suggest distinct trajectories of vascular adaptation after pregnancy, depending on whether the underlying disturbance was limited to angiogenic imbalance or progressed to clinical PE.

### 4.2. Comparison with Other Studies

Previous studies have demonstrated that women with a history of PE exhibit persistent alterations in cardiovascular and metabolic biochemical parameters, including higher blood pressure, dyslipidemia and increased BMI, even decades postpartum [13,19]. However, since most of these studies have been conducted many years after the index pregnancy, the observed biochemical differences may partly reflect the natural progression of cardiovascular risk over time, rather than early post-PE alterations. Evidence has also described higher concentrations of endothelial dysfunction markers decades postpartum, such as soluble vascular cell adhesion molecule-1, soluble E-selectin, sFlt-1, urinary albumin/creatinine ratio, and high-sensitivity C-reactive protein [20,21,22]. However, these studies did not evaluate angiogenic imbalance during pregnancy or its potential association with long-term endothelial dysfunction. In our study, slightly higher levels of proteinuria were observed among women who exhibited angiogenic imbalance during pregnancy, and proteinuria correlated positively with the sFlt-1/PlGF ratio (the higher the ratio, the higher the proteinuria). The exception was the intermediate group (sFlt-1/PlGF between 38 and 85), likely due to the small sample size in this subgroup. The increase in urinary protein may reflect impaired glomerular barrier function, likely secondary to microvascular rarefaction and increased permeability caused by endothelial dysfunction [23]. In line with this, potassium concentrations were also marginally higher in the same group, which could be related to mild tubular dysfunction secondary to the same endothelial injury [24,25]. Both alterations may suggest a low-grade renal involvement, although the differences were small and likely clinically irrelevant.

Elevated serum LDH is a well-recognized marker of hemolysis, tissue injury, and metabolic stress, commonly linked to endothelial damage and microangiopathic processes [26]. In PE, higher LDH levels correlate strongly with disease severity and worse maternal–fetal outcomes [26,27], but no previous studies have analyzed its elevation years after PE. In our study, women with an abnormal sFlt-1/PlGF ratio during pregnancy showed slightly higher LDH concentrations at 3–6 years postpartum. While the mechanisms remain uncertain, this finding may suggest ongoing low-grade tissue injury or metabolic stress, consistent with the systemic effects of chronic endothelial impairment beyond the postpartum period.

Emerging evidence indicates that pregnancies complicated by angiogenic imbalance, such as PE, are associated with persistent epigenetic and transcriptomic reprogramming of maternal monocytes. This reprogramming promotes a shift toward an anti-inflammatory and tolerogenic phenotype, characterized by increased proportions of anti-inflammatory myeloid cells and reduced proportions of inflammatory non-classical monocytes. These changes may result in lower total leukocyte counts and attenuated inflammatory responsiveness that persist long after delivery [28]. Although in our study specific monocyte subsets were not determined, the observed reduction in total leukocyte count should thus be interpreted with caution, although it might be related to this underlying immunological adaptation.

When focusing on the group of women with a history of PE, hs-TnT differed significantly from controls, with higher median levels observed. Previous studies have shown that pregnancies complicated by PE are associated with higher circulating cardiac troponin concentrations compared with normotensive pregnancies [29,30,31]. This elevation has been interpreted as a marker of myocardial stress or subclinical injury, potentially related to the cardiac remodeling and diastolic dysfunction characteristic of PE. A previous study has assessed high-sensitivity troponin I (hs-TnI) a decade after the development of PE showing no differences between women with and without a history of early-onset PE [32]. These discrepancies may reflect methodological and biological differences between troponin assays. Hs-TnI and hs-TnT, although both specific to cardiomyocytes, are distinct proteins with different release kinetics and assay sensitivities. Moreover, hs-TnT has been reported to be more sensitive for detecting chronic low-grade myocardial injury, whereas hs-TnI may be more influenced by acute ischemic events [33]. In our cohort, women with a history of PE had significantly higher hs-TnT concentrations 3–6 years postpartum. This earlier assessment period, together with the use of a highly sensitive assay, may have allowed detection of subtle myocardial stress that could normalize over longer follow-up intervals. These findings may be related with the hypothesis of persistent subclinical cardiac injury after PE and are consistent with previous reports of structural and functional cardiac alterations years after the affected pregnancy [31]. However, the magnitude of this difference was small and is unlikely to be clinically meaningful, especially considering that most women in our cohort were otherwise healthy.

Other studies have shown that women with a history of PE have higher LDL levels compared to women with normotensive pregnancies [34,35]. In our study, LDL concentrations were unexpectedly higher among women without a history of PE. However, this difference appears to be driven by a few outlier values within the control group, as four participants exhibited markedly elevated LDL levels. Moreover, the proportion of participants with diagnosed dyslipidemia did not differ between groups, and the overall lipid profile (including HDL, triglycerides, and total cholesterol) was in fact more favorable among women without previous PE. Therefore, this finding should be interpreted with caution and is unlikely to reflect a true biological difference, but rather random variability related to isolated extreme values.

### 4.3. Clinical Implications

This study provides valuable insights into the underlying mechanisms linking pregnancy-related angiogenic imbalance with later cardiovascular alterations. Beyond these, this study sheds light on a frequently overlooked dimension of women’s health—the kidney. Our results indicate that higher levels of proteinuria and LDH years after delivery may reflect a persistent renal endothelial dysfunction initially triggered by angiogenic imbalance during pregnancy. Such renal involvement often remains silent until advanced stages, meaning that we may be diagnosing these women too late. Our findings support the hypothesis that disrupted angiogenic signaling contributes to long-term endothelial dysfunction. In contrast, women with a clinical history of PE showed higher hs-TnT concentrations but no differences in proteinuria, whereas those with an abnormal sFlt-1/PlGF ratio during pregnancy showed no troponin elevation. This pattern may indicate that the pathways leading to postpartum vascular injury may differ depending on whether the maternal cardiovascular system was sufficiently compromised to develop overt PE during pregnancy. In women who developed PE, myocardial stress may predominate, while in those with subclinical angiogenic imbalance, endothelial dysfunction and low-grade tissue injury may be the main residual processes. From a clinical perspective, these findings emphasize the importance of identifying women at risk already during pregnancy through angiogenic imbalance. Such early identification could guide postpartum cardiovascular monitoring and enable targeted interventions focused on endothelial protection and lifestyle modification, potentially mitigating the long-term cardiovascular burden associated with PE and related placental disorders.

### 4.4. Strengths and Limitations

This study has several strengths. Firstly, it evaluates biochemical and cardiovascular markers 3 to 6 years postpartum in a cohort with comprehensive clinical and biochemical data obtained prospectively during pregnancy, allowing for robust long-term associations. Secondly, it goes beyond previous research focused solely on the clinical diagnosis of PE by integrating angiogenic biomarker data from the index pregnancy with detailed postpartum assessments. Thirdly, participants were extensively characterized during pregnancy, enabling accurate adjustment for potential confounders and identification of early alterations that may precede clinically overt cardiovascular disease. Fourthly, the relatively large and well-defined cohort, together with the inclusion of a broad panel of renal, metabolic, and cardiovascular biomarkers, enhances the reliability and depth of the analyses. Finally, the use of multivariable models adjusting for key confounders reinforces the internal validity and robustness of the findings.

However, this study also has limitations. Firstly, biomarkers were assessed only once at follow-up, precluding evaluation of their temporal evolution. Secondly, gestational age at angiogenic biomarker assessment differed between groups. Nevertheless, the cut-off of 38 is considered valid across all gestational ages, reducing the relevance of this variability. Thirdly, angiogenic biomarker results were not concealed during pregnancy in all cases and could have influenced clinical management, potentially introducing bias through differential treatment or surveillance. In addition, Fourthly, since participants were drawn from previous prospective studies, some degree of selection bias cannot be excluded, as women more engaged with healthcare may have been more likely to attend long-term follow-up visits. Additionally, subsequent pregnancies between the index gestation and follow-up were not excluded; although none were complicated by PE, their potential impact on later cardiovascular health cannot be fully ruled out. Fifth, the differences observed were small and may fall within the normal variability range of laboratory techniques. However, this variability would have affected both groups equally, thereby reinforcing the consistency of our findings despite their limited clinical relevance. Finally, the single-center design and relatively homogeneous study population may limit generalizability to broader settings, and unmeasured factors such as lifestyle, endocrine disorders, socioeconomic background, genetic predisposition, psychosocial stress or other cardiovascular risk factors could act as confounders.

Despite these limitations, our study provides novel insights into the long-term cardiovascular and metabolic profile of women after pregnancy and highlights the potential value of angiogenic biomarkers to identify those who may remain at increased risk several years after delivery. Further research in larger and more varied populations is needed to confirm our findings and to evaluate whether targeted strategies in women with abnormal sFlt-1/PlGF ratios can reduce the progression to overt cardiovascular or renal disease.

## 5. Conclusions

At 3–6 years postpartum, women with a history of PE show slightly higher hs-TnT concentrations, suggesting subtle myocardial stress, whereas those with angiogenic imbalance during pregnancy exhibit higher LDH, potassium, and proteinuria levels, potentially indicating persistent endothelial dysfunction. Although these differences are clinically irrelevant, they may provide insight into underlying pathophysiological mechanisms of long-term cardiovascular involvement after PE. Further studies should evaluate biochemical and cardiovascular parameters later in life to clarify the potential clinical impact of angiogenic imbalance and PE.

## Figures and Tables

**Table 1:** Baseline characteristics of study population according to previous PE.

	No Previous PE (N = 250)	Previous PE (N = 113)	*p* Value
Follow-up interval, years	4.38 (4.04–4.63)	4.38 (3.66–5.10)	0.34
Age at inclusion, years	40.7 (37.3–44.1)	42.3 (38.1–45.0)	0.03
BMI	24.9 (22.2–28.8)	27.7 (24.3–32.8)	<0.01
Race or ethnic group *			0.18
White	188 (75.2)	71 (62.8)	
Black	11 (4.4)	5(4.4)	
Latin American	45 (18.0)	31 (27.4)	
South Asian	2 (0.8)	3 (2.7)	
East Asian	2 (0.8)	1 (0.9)	
Mixed Race	2 (0.8)	2 (1.8)	
**Current Medical History**			
Current Chronic Hypertension	18 (7.2)	23 (20.4)	<0.01
Type 1 or 2 diabetes mellitus	5 (2.0)	5 (4.4)	0.19
Antiphospholipid syndrome	1 (0.4)	0	0.50
Autoimmune disease	11 (4.4)	3 (2.7)	0.42
Dyslipidemia	21 (8.4)	10 (8.9)	0.89
Current Cigarette smoking	5 (2.0)	6 (5.3)	0.09
**Current Arterial Blood Pressure**			
Systolic Blood Pressure, mmHg	110 (103–120)	123 (113–133)	<0.01
Diastolic Blood Pressure, mmHg	72.0 (67.0–79.0)	78.5 (73.0–83.0)	<0.01
Mean Arterial Blood, mmHg	84 (79–93)	93 (86–100)	<0.01
**Pregnancy History**			
Breastfeeding	234 (93.6)	99 (87.6)	0.06
Primiparous	136 (54.4)	64 (56.6)	0.69
Previous spontaneous pregnancy loss	56 (22.4)	29 (25.7)	0.50
Previous Gestational diabetes	31 (12.4)	13 (11.5)	0.79
Pre-existing chronic hypertension	10 (4.0)	11 (9.7)	0.03
Pre-existing type 1 or 2 diabetes mellitus	3 (1.2)	4 (3.5)	0.13
Pre-existing antiphospholipid syndrome	1 (0.4)	0	0.50
Pre-existing autoimmune disease	6 (2.4)	4 (3.5)	0.54
**Angiogenic Biomarkers in Pregnancy**			
sFlt-1 during pregnancy, pg/mL	2396 (1751–3631)	6690 (3836–10,605)	<0.01
PlGF during pregnancy, pg/mL	354 (183–668)	106 (57–184)	<0.01
sFlt-1/PlGF during pregnancy	6.24 (2.76–19.90)	65.20 (25.40–193.00)	<0.01
Gestational age at sFlt-1/PlGF determination, weeks	33.8 (32.0–37.2)	35.6 (33.5–37.6)	0.01
sFlt-1/PlGF < 38	186/214 (86.9)	27/81 (33.3)	<0.01
sFlt-1/PlGF ≥ 38	28/214 (13.1)	54/81 (66.7)	<0.01

Data are given as number (%) or median (IQR). Differences were analysed using chi-square test for categorical variables and independent-sample Student’s *t*-test or Mann–Whitney U-test, as appropriate, for continuous variables. * Race and ethnicity were self-reported by participants from predefined categories. BMI, body mass index, PlGF, placental growth factor; sFlt-1, soluble fms-like tyrosine kinase-1, PE, preeclampsia.

**Table 2:** Hematological, biochemical and cardiovascular biomarker concentrations according to previous PE.

Hematological and Biochemical Parameters	No Previous PE (n = 250)	Previous PE (n = 113)	Adjusted *p* Value *
Hemoglobin, g/L	130 (122–136)	131 (121–137)	0.53
Hematocrit, l/L	0.39 (0.37–0.41)	0.39 (0.37–0.41)	0.60
Leukocytes, U/mL	6205 (5300–5570)	6310 (5570–7380)	0.94
Platelets, U/mL	262,500 (222,250–309,750)	279,000 (243,000–313,000)	0.12
Glucose, mg/dL	86.5 (82.0–92.0)	89.0 (83.0–94.0)	0.59
Sodium, mmol/L	139 (138–141)	140 (138–141)	0.25
Potassium, mmol/L	4.22 (4.04–4.39)	4.23 (4.01–4.37)	0.91
Uric acid, mg/dL	4.07 (3.51–4.65)	4.03 (3.39–4.69)	0.68
Creatinine, mg/dL	0.67 (0.62–0.74)	0.65 (0.59–0.71)	0.09
Glycated hemoglobin, %	5.3 (5.2–5.5)	5.4 (5.3–5.6)	0.25
AST, U/L	20.0 (17.0–23.8)	20.0 (17.0–24.0)	0.19
ALT, U/L	15 (12–20)	16 (12–23)	0.32
Bilirubin, mg/dL	0.59 (0.45–0.81)	0.54 (0.43–0.67)	0.05
LDH, U/L	162 (148–179)	166 (152–187)	0.36
LDL, mg/dL	111.0 (93.3–132.0)	104.0 (93.0–130.0)	0.03
VLDL, mg/dL	11.7 (9.1–16.2)	14.3 (10.3–20.3)	0.76
HDL, mg/dL	59.6 (50.0–68.4)	55.7 (46.4–65.8)	0.74
Cholesterol, mg/dL	184 (166–207)	179 (162–207)	0.11
Triglycerides, mg/dL	58.1 (48.7–80.7)	70.8 (51.3–101.0)	0.86
Urinary Protein, g/L	0.08 (0.07–0.13)	0.08 (0.07–0.12)	0.46
Urinary Albumin, mg/L	8.4 (5.0–14.7)	8.0 (5.1–13.8)	0.09
TSH, mUI/L	1.33 (1.04–1.96)	1.41 (1.11–1.65)	0.26
Prolactin, mUI/L	260 (210–449)	309 (214–367)	0.94
**Cardiovascular biomarkers**	**No previous PE** **(n = 163)**	**Previous PE** **(n = 66)**	Adjusted *p* value *
PlGF, pg/mL	10.00 (9.00–12.00)	11.00 (9.68–12.00)	0.46
NT-ProBNP, ng/L	42.2 (25.4–66.0)	46.0 (26.0–76.2)	0.36
Hs-TnT, ng/L	3.2 (3.0–5.0)	4.0 (3.0–6.0)	0.03

Data are given as median (IQR). * Differences were analyzed using linear regression methods adjusted for Age, BMI, and Mean arterial Blood pressure. AST, aspartate aminotransferase, ALT, alanine aminotransferase, LDL, low-density lipoprotein, LDH, lactate dehydrogenase, VLDL, very low-density lipoprotein, HDL, high-density lipoprotein, TSH, thyroid-stimulating hormone, PlGF, placental growth factor, NT-ProBNP, N-terminal pro-brain natriuretic peptide, hs-TnT, high soluble troponin T.

**Table 3:** Baseline characteristics of study population according to sFlt-1/PlGF ratio, regardless of previous PE.

	sFlt-1/PlGF < 38 (n = 213)	sFlt-1/PlGF ≥ 38 (n = 82)	*p* Value
Follow-up interval, years	4.33 (3.93–4.60)	4.38 (3.65–5.00)	0.18
Age at inclusion, years	40.2 (37.0–44.3)	41.6 (37.8–44.4)	0.33
BMI	24.8 (22.2–29.4)	28.3 (24.6–32.7)	<0.01
Race or ethnic group *			0.22
White	160 (75.2)	54 (65.9)	
Black	11 (5.2)	2 (2.4)	
Latin American	35 (16.4)	23 (28.1)	
South Asian	3 (1.4)	2 (2.4)	
East Asian	2 (0.9)	0	
Mixed Race	2 (0.9)	1 (1.2)	
**Current Medical History**			
Current chronic hypertension	20 (9.4)	19 (23.2)	<0.01
Type 1 or 2 diabetes mellitus	4 (1.9)	4 (4.9)	0.16
Antiphospholipid syndrome	1 (0.5)	0	0.53
Autoimmune disease	13 (6.1)	5 (6.1)	0.59
Dyslipidemia	18 (8.5)	9 (11.0)	0.50
Current cigarette smoking	8 (3.8)	2 (2.4)	0.58
**Current Arterial Blood Pressure**			
Systolic Blood Pressure, mmHg	111 (103–121)	122 (113–130)	<0.01
Diastolic Blood Pressure, mmHg	72.5 (67.0–81.0)	77.5 (71.3–82.0)	<0.01
Mean Arterial Blood, mmHg	85.0 (79.0–94.0)	92.0 (87.0–98.8)	<0.01
**Pregnancy history**			
Breastfeeding	200 (93.9)	72 (87.8)	0.08
Primiparous	108 (50.7)	53 (64.6)	0.03
Previous spontaneous pregnancy loss	45 (21.1)	16 (19.5)	0.76
Previous gestational diabetes	28 (13.2)	7 (8.5)	0.27
Pre-existing chronic hypertension	11 (5.2)	10 (12.2)	0.04
Pre-existing type 1 or 2 diabetes mellitus	2 (0.9)	3 (3.7)	0.11
Pre-existing antiphospholipid syndrome	1 (0.5)	0	0.53
Pre-existing autoimmune disease	8 (3.8)	1 (1.2)	0.26
**Angiogenic Biomarkers in Pregnancy**			
sFlt-1 during pregnancy, pg/ml	2273 (1729–3156)	8619 (5895–11,495)	<0.01
PlGF during pregnancy, pg/ml	417.0 (219.0–687.0)	75.0 (47.3–112.0)	<0.01
sFlt-1/PlGF during pregnancy, pg/ml	5.56 (2.61–14.00)	88.30 (60.00–213.00)	<0.01
Gestational age at sFlt-1/PlGF determination, weeks	33.3 (31.8–36.2)	37.0 (34.6–38.3)	<0.01

Data are given as number (%) or median (IQR). * Differences were analyzed using chi-square test for categorical variables and independent-sample Student’s *t*-test or Mann–Whitney U-test, as appropriate, for continuous variables. * Race and ethnicity were self-reported by participants from predefined categories. BMI, body mass index, PlGF, placental growth factor; sFlt-1, soluble fms-like tyrosine kinase-1, FGR, fetal growth restriction; PE, preeclampsia.

**Table 4:** Hematological, biochemical and cardiovascular biomarkers concentrations according to sFlt-1/PlGF ratio, regardless of previous PE.

Hematological and Biochemical Parameters	sFlt-1/PlGF < 38 (n = 213)	sFlt-1/PlGF ≥ 38 (n = 82)	Adjusted *p* Value *
Hemoglobin, g/L	130 (122–137)	132 (121–136)	0.68
Hematocrit, l/L	0.390 (0.370–0.400)	0.390 (0.370–0.407)	0.08
Leukocytes, U/ml	6250 (5430–7450)	6150 (5348–7055)	0.03
Platelets, U/mcL	264,000 (222,000–313,000)	279,000 (222,000–3,077,501)	0.74
Glucose, mg/dL	86 (82–91)	89 (83–94)	0.87
Sodium, mmol/L	139 (138–141)	139 (138–140)	0.97
Potassium, mmol/L	4.19 (4.02–4.37)	4.25 (4.07–4.40)	0.048
Uric acid, mg/dL	4.08 (3.53–4.69)	3.95 (3.38–4.72)	0.84
Creatinine, mg/dL	0.67 (0.62–0.74)	0.65 (0.60–0.74)	0.84
Glycated hemoglobin, %	5.3 (5.2–5.5)	5.4 (5.3–5.6)	0.26
AST, U/L	20 (17–23)	20 (17–23)	0.32
ALT, U/L	15.0 (12.0–21.0)	16.0 (12.0–22.8)	0.58
Bilirubin, mg/dL	0.570 (0.450–0.780)	0.560 (0.443–0.728)	0.84
LDH, U/L	163 (149–177)	168 (150–189)	0.046
LDL, mg/dL	110.0 (93.4–13.2)	109.0 (93.6–134.0)	0.40
VLDL, mg/dL	12.50 (9.27–18.20)	13.50 (9.81–18.30)	0.14
HDL, mg/dL	59.2 (49.5–68.1)	57.1 (46.5–66.3)	0.96
Cholesterol, mg/dL	183 (166–208)	182 (166–208)	0.26
Triglycerides, mg/dL	62.0 (48.7–90.3)	66.8 (50.0–90.9)	0.15
Urinary Protein, g/L	0.08 (0.07–0.13)	0.09 (0.07–0.18)	0.01
Urinary Albumin, mg/L	8.7 (5.2–15.5)	7.3 (5.0–13.3)	0.11
TSH, mUI/L	1.33 (1.02–1.67)	1.40 (1.18–1.90)	0.88
Prolactin, mUI/L	260 (217–415)	309 (219–348)	0.78
**Cardiovascular Biomarkers**	**sFlt-1/PlGF < 38** **(n = 171)**	**sFlt-1/PlGF > 38** **(n = 58)**	Adjusted *p* *
PlGF, pg/mL	10.0 (9.0–12.0)	11.0 (9.5–12.0)	0.91
NT-ProBNP, ng/L	45.0 (25.5–69.0)	47.0 (26.5–78.5)	0.14
Hs-TnT, ng/L	3.5 (3.0–5.0)	4.0 (3.0–6.0)	0.22

Data are given as median (IQR). * Differences were analyzed using linear regression methods adjusted for potential cofounding factors such as BMI, Parity and Mean Arterial Blood pressure. AST, aspartate aminotransferase, ALT, alanine aminotransferase, LDL, low-density lipoprotein, LDH, lactate dehydrogenase, VLDL, very low-density lipoprotein, HDL, high-density lipoprotein, TSH, thyroid-stimulating hormone, PlGF, placental growth factor, NT-ProBNP, N-terminal pro-brain natriuretic peptide, hs-TnT, high soluble troponin T.

## Data Availability

The original contributions presented in this study are included in the article/Supplementary Material. Further inquiries can be directed to the corresponding author.

## Supplementary Materials

The following supporting information can be downloaded at: https://www.mdpi.com/article/10.3390/jcm14238389/s1, Table S1: Baseline characteristics of study population according to previous early or late-onset PE, Table S2: Hematological and biochemical measurements according to previous early or late-onset PE, Table S3: Baseline characteristics of study population according to different sFlt-1/PlGF cut-offs; Table S4: Hematological, biochemical and cardiovascular biomarkers measurements according to different sFlt-1/PlGF cut-offs, Table S5: Reference ranges of hematological and biochemical parameters and cardiovascular biomarkers.

## Abbreviations

PE	Preeclampsia
sFlt-1	Soluble fms-like tyrosine kinase-1
PlGF	Placental growth factor
TSH	Thyroid stimulating hormone
NT-proBNP	N-terminal pro B-type natriuretic peptide
Hs-TnT	High sensitivity Troponin T
IQRs	Interquartile ranges
STROBE	Strengthening the reporting of observational studies in epidemiology
BMI	Body mass index
AST	Aspartate aminotransferase
ALT	Alanine aminotransferase
LDL	Low-density lipoprotein
LDH	Lactate dehydrogenase
VLDL	Very low-density lipoprotein
HDL	High-density lipoprotein
sVCAM-1	Soluble vascular cell adhesion molecule 1
hsCRP	High sensitivity C-reactive protein
Hs-TNI	High sensitivity Troponin I
