# Supplementary material for: Long-Term Biochemical and Cardiovascular Profiles 3–6 Years After Preeclampsia: Impact of Angiogenic Imbalance During Pregnancy
Source: J Clin Med. 2025 Nov 26;14(23):8389. doi: 10.3390/jcm14238389 (PMC12692977; doi:10.3390/jcm14238389)
Supplement: Supplementary file 1 [file jcm-14-08389-s001.zip › jcm-3997762-supplementary.pdf]

*Table S1: Baseline characteristics of study population according to previous early or late-onset PE*

| | No previous PE (N=250) | Previous early-onset PE (N=41) | Previous late-onset PE (N=72) | P value |
|---|---|---|---|---|
| Follow-up interval, years | 4.38 (4.04-4.63) | 4.17 (3.30-4.92) | 4.50 (4.05-5.11) | 0.07 |
| Age at inclusion, years | 40.7 (37.3-44.1) | 41.5 (38.6-44.0) | 42.4 (37.9-45.5) | 0.11 |
| BMI | 24.9 (22.2-28.8) | 28.2 (23.9-35.3)[a] | 27.3 (24.6-31.5)[a] | <0.01 |
| Race or ethnic group* | | | [a] | 0.03 |
| White | 188 (75.2) | 27 (65.9) | 44 (61.1) | |
| Black | 11 (4.4) | 3 (7.3) | 2 (2.8) | |
| Latin American | 45 (18.0) | 8 (19.5) | 23 (31.9) | |
| South Asian | 2 (0.8) | 1 (2.4) | 2 (2.8) | |
| East Asian | 2 (0.8) | 0 | 1 (1.4) | |
| Mixed Race | 2 (0.8) | 2 (4.9) | 0 | |
| **Current Medical History** | | | | |
| Current Chronic Hypertension | 18 (7.2) | 11 (26.8)[a] | 12 (17.0)[a] | <0.01 |
| Type 1 or 2 diabetes mellitus | 5 (2.0) | 3 (7.3) | 2 (2.8) | 0.16 |
| Antiphospholipid syndrome | 1 (0.4) | 0 | 0 | 0.80 |
| Autoimmune disease | 11 (4.4) | 2 (4.9) | 1 (1.4) | 0.47 |
| Dyslipidemia | 21 (8.4) | 5 (12.2) | 5 (6.9) | 0.63 |
| Current cigarette smoking | 5 (2.0) | 2 (4.9) | 4 (5.6) | 0.24 |
| **Current Arterial Blood Pressure** | | | | |
| Systolic Blood Pressure, mmHg | 110 (103-120) | 127 (116-138)[a] | 121 (113-133)[a] | <0.01 |
| Diastolic Blood Pressure, mmHg | 72 (67-79) | 81 (73-83)[a] | 77 (73-82)[a] | <0.01 |
| Mean Arterial Blood, mmHg | 84 (79-93) | 96 (87-102)[a] | 92 (86-100)[a] | <0.01 |
| **Pregnancy History** | | | | |
| Breastfeeding | 234 (93.6) | 34 (82.9) | 65 (90.3) | 0.06 |
| Primiparous | 136 (54.4) | 20 (48.8) | 28 (38.9) | 0.42 |
| Previous spontaneous pregnancy loss | 56 (22.4) | 9 (22.0) | 20 (33.3) | 0.62 |
| Previous Gestational diabetes | 31 (12.4) | 6 (14.6) | 7 (9.7) | 0.72 |
| Pre-existing chronic hypertension | 10 (4.0) | 6 (14.6)[a] | 5 (4.2) | 0.02 |
| Pre-existing type 1 or 2 diabetes mellitus | 3 (1.2) | 2 (4.9) | 2 (2.8) | 0.24 |
| Pre-existing antiphospholipid syndrome | 1 (0.4) | 0 | 0 | 0.80 |
| Pre-existing autoimmune disease | 6 (2.4) | 2 (4.9) | 2 (2.8) | 0.67 |
| **Angiogenic Biomarkers in Pregnancy** | | | | |
| sFlt-1 during pregnancy, pg/ml | 2396 (1751-3631) | 8449 (3180-13537)[a] | 6651 (4434-9915)[a] | <0.01 |

| | | | | |
|---|---|---|---|---|
| PlGF during pregnancy, pg/ml | 354.0 (183.0-668.0) | 75.0 (42.5-230.0)[a] | 108.5 (73.0-173.0)[a] | <0.01 |
| sFlt-1/PlGF during pregnancy, pg/ml | 6.24 (2.76-19.90) | 133.00(12.00 -328.00)[a] | 61.50 (29.40-101.00)[a] | <0.01 |
| Gestational age at sFlt-1/PlGF determination, weeks | 33.8 (32.0-37.2) | 34.0 (32.5-36.0) | 36.5 (35.0-38.2)[a,b] | <0.01 |

Data are given as number of events (%) or median (IQR). Differences were analysed using the Krustal-Wallis test and the Dwass-Steel-Critchlow-Fligner multiple comparison *post-hoc* test.

[a], significant difference vs no previous PE group

[b], significant difference vs previous early-onset

*Race and ethnicity were self-reported by participants from predefined categories. BMI, body mass index, PlGF, placental growth factor; sFlt-1, soluble fms-like tyrosine kinase-1, FGR, fetal growth restriction; PE, preeclampsia

*Table S2: Hematological and biochemical measurements according to previous early or late-onset PE.*

| Hematological and biochemical parameters | No previous PE (n=250) | Previous early-onset PE (N=41) | Previous late-onset PE (N=72) | Adjusted p value* |
|---|---|---|---|---|
| Haemoglobin, g/l | 130 (122-137) | 131 (121-137) | 131 (121-137) | 0.68 |
| Haematocrit, l/l | 0.39 (0.37-0.40) | 0.39 (0.36-0.41) | 0.39 (0.37-0.40) | 0.66 |
| Leukocytes, U/ml | 6250 (5430-7450) | 6460 (5800-7320) | 6260 (5198-7523) | 0.77 |
| Platelets, U/mcl | 264000 (222000-313000) | 279000 (253000-321000) | 281000 (235750-308750) | 0.45 |
| Glucose, mg/dl | 86.0 (82.0-91.0) | 80.0 (80.0-94.0) | 89.5 (84.0-93.0) | 0.87 |
| Sodium, mmol/l | 139 (138-141) | 139 (138-140) | 140 (139-141) | 0.11 |
| Potassium, mmol/l | 4.19 (4.02-4.37) | 4.19 (4.03-4.32) | 4.24 (4.01-4.38) | 0.83 |
| Uric acid, mg/dl | 4.08 (3.53-4.69) | 4.30 (3.40-5.00) | 3.90 (3.40-4.60) | 0.71 |
| Creatinine, mg/dl | 0.670 (0.620-0.740) | 0.665 (0.600-0.725) | 0.650(0.590-0.700) | 0.20 |
| Glycated haemoglobin, % | 5.3 (5.2-5.5) | 5.4 (5.3-5.7) | 5.4 (5.3-5.6) | 0.43 |
| AST, U/l | 20 (17-23) | 20.0 (17.0-23.0) | 20.5 (17.0-24.0) | 0.34 |
| ALT, U/l | 15.0 (12.0-21.0) | 16.0 (11.0-21.0) | 16.0 (12.8-24.0) | 0.64 |
| Bilirubin, mg/dl | 0.57 (0.45-0.78) | 0.56 (0.49-0.66) | 0.54 (0.41-0.67) | 0.06 |
| LDH, U/l | 163 (149-177) | 164 (153-188) | 168 (152-186) | 0.44 |
| LDL, mg/dl | 110.0 (93.4-13.2) | 105.0 (90.5-133.0) | 104.0 (94.1-124.0) | 0.16 |
| VLDL, mg/dl | 12.50 (9.27-18.20) | 13.60 (10.30-20.30) | 14.40 (10.60-21.20) | 0.64 |
| HDL, mg/dl | 59.2 (49.5-68.1) | 53.8 (45.3-66.6) | 55.9 (48.3-64.7) | 0.68 |
| Cholesterol, mg/dl | 183 (166-208) | 178 (163-209) | 180 (162-201) | 0.43 |
| Triglycerides, mg/dl | 62.0 (48.7-90.3) | 67.3 (51.3-101.0) | 71.2 (52.4-105.0) | 0.74 |
| Protein, g/l | 0.080 (0.070-0.130) | 0.090 (0.070-0.185) | 0.080 (0.070-0.120) | 0.53 |
| Albumin, mg/l | 8.70 (5.20-15.50) | 9.50 (5.88-19.40) | 7.50 (5.00-12.40) | 0.10 |
| TSH, mUI/l | 1.33 (1.02-1.67) | 1.39 (0,84-1.41) | 1.66 (1.51-1.95) | 0.57 |
| Prolactin, mUI/l | 260 (217-415) | 218 (202-270) | 348 (348-421) | 0.79 |
| **Cardiovascular biomarkers** | **No previous PE (n=163)** | **Previous early-onset PE (n=16)** | **Previous late-onset PE (n= 50)** | **Adjusted p*** |
| PlGF, pg/ml | 10.0 (9.0-12.0) | 11.0 (10.0-12.0) | 11.0 (9.0-12.0) | 0.57 |
| NT-ProBNP, ng/l | 45.0 (25.5-69.0) | 38.0 (20.5-74.6) | 47.5 (35.2-78.8) | 0.16 |
| Hs-TnT, ng/l | 3.5 (3.0-5.0) | 4.00 (3.00-6.00) | 3.56 (3.00-5.00) | 0.06 |

Data are given as median (IQR).

*Differences were analysed using linear regression methods adjusted for potential cofounding factors such as BMI, Race and Mean Arterial Blood pressure.

AST, aspartate aminotransferase, ALT, alanine aminotransferase, LDL, low-density lipoprotein, LDH, lactate dehydrogenase, VLDL, very low-density lipoprotein, HDL, high-density lipoprotein, TSH, thyroid-stimulating hormone, PlGF, placental growth factor, NT-ProBNP, N-terminal pro-brain natriuretic peptide, hs-TnT, high soluble troponin T.

*Table S3: Baseline characteristics of study population according to different sFlt-1/PlGF ratio cut-offs*

| | **sFlt-1/PlGF<38 (n=213)** | **sFlt-1/PlGF ≥38 <85 (n=39)** | **sFlt-1/PlGF ≥85 <110 (n=8)** | **sFlt-1/PlGF ≥110 (n=35)** | **p-value** |
|---|---|---|---|---|---|
| Follow-up interval, years | 4.33 (3.93-4.60) | 4.39 (3.83-4.78) | 4.49 (4.15-5.04) | 4.34 (3.51-5.09) | 0.53 |
| Age at inclusion, years | 40.2 (37.0-44.3) | 41.7 (37.8-45.4) | 39.1 (32.4-43.0) | 41.5 (38.3-44.2) | 0.27 |
| BMI | 24.8 (22.2-29.4) | 27.8 (25.0-30.8)[a] | 27.3 (24.5-31.2) | 31.1 (24.1-34.7)[a] | <0.01 |
| Race or ethnic group* | | | | [a] | 0.11 |
| White | 160 (75.2) | 29 (74.4) | 5 (62.5) | 20 (57.1) | |
| Black | 11 (5.2) | 1 (2.6) | 1 (12.5) | 0 | |
| Latin American | 35 (16.4) | 8 (20.5) | 2 (25.0) | 13 (37.1) | |
| South Asian | 3 (1.4) | 1 (2.6) | 0 | 1 (2.9) | |
| East Asian | 2 (0.9) | 0 | 0 | 0 | |
| Mixed Race | 2 (0.9) | 0 | 0 | 1 (2.9) | |
| **Current Medical History** | | | | | |
| Current chronic hypertension | 20 (9.4) | 10 (25.6)[a] | 2 (25.0) | 7 (20.0) | 0.02 |
| Type 1 or 2 diabetes mellitus | 4 (1.9) | 1 (2.6) | 1 (12.5)[a] | 2 (5.7) | 0.20 |
| Antiphospholipid syndrome | 1 (0.5) | 0 | 0 | 0 | 0.94 |
| Autoimmune disease | 13 (6.1) | 2 (5.1) | 0 | 1 (2.9) | 0.85 |
| Dyslipidemia | 18 (8.5) | 3 (7.7) | 1 (12.5) | 5 (14.3) | 0.70 |
| Current cigarette smoking | 8 (3.8) | 2 (5.1) | 0 | 0 | 0.58 |
| **Current Arterial Blood Pressure** | | | | | |
| Systolic Blood Pressure, mmHg | 111 (103-121) | 122 (114-131)[a] | 127 (117-133)[a] | 120 (114-129)[a] | <0.01 |
| Diastolic Blood Pressure, mmHg | 72.5 (67.0-81.0) | 76 (72-80.5)[a] | 75 (72.5-80.5) | 79 (69.5-83) | 0.01 |
| Mean Arterial Blood, mmHg | 85.0 (79.0-94.0) | 91.7 (87.3-97.5)[a] | 90.7 (88.3-95.6) | 93.0 (85.0-99.2)[a] | <0.01 |
| **Pregnancy History** | | | | | |
| Breastfeeding | 200 (93.9) | 34 (87.2) | 8 (100.0) | 30 (85.7) | 0.18 |
| Primiparous | 108 (50.7) | 23 (59.0) | 6 (75.0) | 24 (68.6) | 0.13 |
| Previous spontaneous pregnancy loss | 45 (21.1) | 11 (28.2) | 1 (12.5) | 4 (11.4) | 0.32 |
| Previous gestational diabetes | 28 (13.2) | 2 (5.1) | 1 (12.5) | 4 (11.4) | 0.56 |
| Pre-existing chronic hypertension | 11 (5.2) | 6 (15.4) | 0 | 4 (11.4) | 0.08 |
| Pre-existing type 1 or 2 diabetes mellitus | 2 (0.9) | 1 (2.6) | 1 (12.5)[a] | 1 (2.9) | 0.08 |
| Pre-existing antiphospholipid syndrome | 1 (0.5) | 0 | 0 | 0 | 0.94 |

| | | | | | |
|---|---|---|---|---|---|
| Pre-existing autoimmune disease | 8 (3.8) | 0 | 0 | 1 (2.9) | 0.61 |
| **Angiogenic Biomarkers in Pregnancy** | | | | | |
| sFlt-1 during pregnancy, pg/ml | 2273 (1729-3156) | 6511 (4773-7690)[a.b] | 9655 (6656-10714)[a] | 11690 (9547-15781)[a] | <0.01 |
| PlGF during pregnancy, pg/ml | 417.0 (219.0-687.0) | 108.0(78.0-150.0)[a.b] | 95.5 (70.0-116.0)[a.b] | 46.0 (34.5-61.5)[a] | <0.01 |
| sFlt-1/PlGF during pregnancy, pg/ml | 5.56 (2.61-14.00) | 59.50 (48.30-68.40)[a.b.c] | 94.80 (88.50-103.00)[a.b] | 236.00 (184.00-391.00)[a] | <0.01 |
| Gestational age at sFlt-1/PlGF determination, weeks | 33.3 (31.8-36.2) | 38.0 (36.0-39.0)[a.b] | 37.1 (36.3-38.5)[a] | 34.3 (33.1-37.2) | <0.01 |

Data are given as number of events (%) or median (IQR). Differences were analyzed using the Krustal-Wallis test and the Dwass-Steel-Critchlow-Fligner multiple comparison *post-hoc* test.

[a], significant difference vs sFlt-1/PlGF ratio <38 group

[b], significant difference vs sFlt-1/PlGF ratio >110

[c], significant difference vs sFlt-1/PlGF ratio >85 and <110

*Race and ethnicity were self-reported by participants from predefined categories.

BMI, body mass index, PlGF, placental growth factor; sFlt-1, soluble fms-like tyrosine kinase-1, FGR, fetal growth restriction; PE, preeclampsia

*Table S4: Hematological, biochemical and cardiovascular biomarkers measurements according to different sFlt-1/PlGF ratio cut-offs*

| Hematological and biochemical parameters | sFlt-1/PlGF<38 (n=213) | sFlt-1/PlGF≥38 <85 (n=39) | sFlt-1/PlGF≥85 <110 (n=8) | sFlt-1/PlGF≥110 (n=35) | Adjusted p value* |
|---|---|---|---|---|---|
| Hemoglobin, g/l | 130 (122-137) | 130 (121-135) | 135 (124-142) | 132 (126-139) | 0.53 |
| Hematocrit, l/l | 0.390 (0.370-0.400) | 0.390 (0.370-0.400) | 0.390 (0.373-0.422) | 0.390 (0.375-0.415)[b] | <0.01 |
| Leukocytes, U/ml | 6250 (5430-7450) | 5870 (5365-6470) | 6550 (6180-7148) | 6450 (5200-7285) | 0.18 |
| Platelets, U/ml | 264000 (221500-312500) | 279000 (227000-304500) | 274500 (233250-301750) | 283000 (239500-319500) | 0.58 |
| Glucose, mg/dl | 86.0 (82.0-91.0) | 89.0 (83.0-95.0) | 89.5 (87.3-97.5) | 89.0 (81.5-93.0) | 0.62 |
| Sodium, mmol/l | 139 (138-141) | 139 (138-141) | 139 (139-139) | 1401 (138-140) | 0.78 |
| Potassium, mmol/l | 4.19 (4.02-4.37) | 4.29 (4.10-4.34) | 4.34 (4.18-4.42) | 4.23 (4.08-4.38) | 0.13 |
| Uric acid, mg/dl | 4.08 (3.53-4.69) | 3.70 (3.36-4.37) | 3.79 (3.65-4.17) | 4.31 (3.35-5.08) | 0.24 |
| Creatinine, mg/dl | 0.670 (0.620-0.740) | 0.650 (0.590-0.750) | 0.650 (0.597-0.702) | 0.650 (0.610-0.737) | 0.82 |
| Glycated hemoglobin, % | 5.30 (5.20-5.50) | 5.40 (5.30-5.50) | 5.55 (5.38-5.73) | 5.40 (5.25-5.75) | 0.27 |
| AST, U/l | 20.0 (17.0-23.0) | 20.0 (17.0-23.0) | 19.0 (17.8-21.3) | 20.0 (17.5-24.5) | 0.39 |
| ALT, U/l | 15.0 (12.0-21.0) | 15.0 (12.5-21.0) | 13.5 (10.8-25.3) | 18.0 (13.0-24.0) | 0.73 |
| Bilirubin, mg/dl | 0.570 (0.450-0.780) | 0.560 (0.430-0.730) | 0.500 (0.470-0.578) | 0.600 (0.450-0.730) | 0.77 |
| LDH, U/l | 163 (149-177) | 175 (152-188) | 155 (145-164) | 168 (151-202) | 0.06 |
| LDL, mg/dl | 110.0 (93.4-132.0) | 114.0 (94.9-136.0) | 98.0 (79.3-113.0) | 104.0 (91.4-137.0) | 0.27 |
| VLDL, mg/dl | 12.50 (9.27-18.20) | 13.60 (9.81-16.10) | 16.00 (9.86-27.40) | 13.40 (8.74-18.70) | 0.29 |
| HDL, mg/dl | 59.2 (49.5-68.1) | 56.1 (49.2-62.9) | 61.9 (55.9-67.0) | 53.0 (45.3-68.7) | 0.59 |
| Cholesterol, mg/dl | 183 (166-208) | 183 (164-209) | 179 (173-182) | 182 (168-209) | 0.28 |
| Triglycerides, mg/dl | 62.0 (48.7-90.3) | 67.3 (50.0-79.7) | 79.2 (48.9-136.0) | 66.4 (50.0-92.9) | 0.33 |
| Protein, g/l | 0.080 (0.070-0.130) | 0.085 (0.070-0.130)[a] | 0.070 (0.070-0.095) | 0.110 (0.070-0.372)[a] | 0.03 |
| Albumin, mg/l | 8.60 (5.20-15.50) | 6.40 (5.00-12.60) | 5.00 (5.00-8.23) | 8.90 (5.05-17.50) | 0.24 |
| TSH, mUI/l | 1.33 (1.02-1.67) | 2.79 (2.79-2.79) | 1.39 (1.39-1.39) | 1.26 (1.04-1.57) | 0.49 |
| Prolactin, mUI/l | 260 (217-415) | 347 (347-347) | 138 (138-138) | 309 (253-420) | 0.97 |

| Cardiovascular biomarkers | sFlt-1/PlGF<38 (n=171) | sFlt-1/PlGF≥38 <85 (n=25) | sFlt-1/PlGF≥85 <110 (n=6) | sFlt-1/PlGF≥110 (n=27) | Adjusted p value* |
|---|---|---|---|---|---|
| PlGF, pg/ml | 10.0 (9.0-12.0) | 11.0 (9.0-12.0) | 11.0 (10.0-12.8) | 11.0 (10.0-11.6) | 0.97 |
| NT-ProBNP, ng/l | 45.0 (25.5-69.0) | 45.5 (29.0-64.8) | 48.0 (33.5-87.3) | 40.0 (22.5-79.5) | 0.15 |
| Hs-TnT, ng/l | 3.5 (3.0-5.0) | 4.0 (3.0-5.0) | 4.0 (3.0-6.5) | 5.0 (3.0-6.0) | 0.08 |

Data are given as median (IQR).

*Differences were analyzed using linear regression methods adjusted for potential cofounding factors such as BMI, and Mean Arterial Blood pressure.

[a], significant difference vs sFlt-1/PlGF ratio <38 group, p<0.05;

[b], significant difference vs sFlt-1/PlGF ratio <38 group, p<0.01.

AST, aspartate aminotransferase, ALT, alanine aminotransferase, LDL, low-density lipoprotein, LDH, lactate dehydrogenase, VLDL, very low-density lipoprotein, HDL, high-density lipoprotein, TSH, thyroid-stimulating hormone, PlGF, placental growth factor, NT-ProBNP, N-terminal pro-brain natriuretic peptide, hs-TnT, high soluble troponin T.

*Table S5: Reference ranges of hematological and biochemical parameters and cardiovascular biomarkers*

| **Hematological and biochemical parameters** | **Normal values** |
|---|---|
| Hemoglobin, g/l | 120-150 |
| Hematocrit, l/l | 0.35-0.45 |
| Leukocytes, U/ml | 3800-11000 |
| Platelets, U/ml | 140000-350000 |
| Glucose, mg/dl | 54-108 |
| Sodium, mmol/l | 136-145 |
| Potassium, mmol/l | 3.5-5.1 |
| Uric acid, mg/dl | 2.5-2.9 |
| Creatinine, mg/dl | 0.57-1.11 |
| Glycated hemoglobin, % | 4.6-5.8 |
| AST, U/l | 5-34 |
| ALT, U/l | 0-30 |
| Bilirubin, mg/dl | 0.29-1.17 |
| LDL, mg/dl | <116 |
| LDH, U/l | 125-220 |
| VLDL, mg/dl | <3.1 |
| HDL, mg/dl | >38.7 |
| Cholesterol, mg/dl | <193 |
| Triglycerides, mg/dl | <151 |
| Protein, g/l | 0-0.12 |
| Albumin, mg/l | 0.1-20.0 |
| TSH, mUI/l | 0.3-5.0 |
| Prolactin, mUI/l | 102-496 |
| **Cardiovascular biomarkers** | |
| NT-ProBNP, ng/l | >300 |
| Hs-TnT, ng/l | <13 |
